# Representations of female characters in Bollywood cinema: stereotypes, audience perceptions, and societal impacts

**DOI:** 10.3389/fsoc.2025.1694300

**Published:** 2026-02-12

**Authors:** Forhana Choudhury, Swati Sharma

**Affiliations:** School of Social Sciences and Languages, Vellore Institute of Technology, Chennai, India

**Keywords:** audience perception, binary representation of women, Bollywood cinema, gender stereotypes, item songs, Madonna-Whore dichotomy, male gaze, sexual objectification

## Abstract

**Introduction:**

The study aims to examine how audiences perceive the contrasting portrayals of the female lead (heroine) and the item girl in Bollywood films, and to analyse how these binary representations reflect attitudes toward women's roles and identities. Grounded in the Reinforcement Theory of Motivation, the research investigates how repeated reward—punishment cues in these portrayals influence audience's interpretations and internalization of binary representations of female characters in Bollywood films.

**Methodology:**

For the study, a quantitative survey was conducted among 300 young respondents across multiple Indian states and NRI audiences. The study specifically examines audience perceptions of the heroine vs. the item girl in Bollywood, using a structured questionnaire, analyzed through ANOVA, chi-square, correlation, and regression analyses. These statistical results were further interpreted through qualitative theoretical lenses of the Reinforcement Theory of Motivation and the Madonna-Whore Dichotomy, enabling a theory-driven understanding of how audiences internalize these gendered portrayals.

**Results:**

Results elucidate a clear distinction in how audiences perceive the heroine and the item girl, with the heroine associated with virtue, respectability, and relatability, while the item girl is marked by hyper-sexualization and social deviance. Regression analyse showed that only the portrayal of the item girl and women's real-life relatabilitywere significant predictors of behavioral perceptions, while commonality and the perception that women are shown differently onscreen were not significant predictors. Meanwhile, correlation analyse indicates the persistence of compartmentalized gender binaries.

**Discussion:**

The outcomes illuminate how Bollywood songs not only reproduce gender stereotypes but also disseminate them through widespread digital circulation, intensifying their impact on young audiences. Moreover, the paper examines how Bollywood's heroine—item girl divide contributes to shaping gender norms and further restricting women's identities in Bollywood cinema.

## 1 Introduction

Bollywood is one of the most powerful cultural spaces in South Asia (Smith, 2017). It has long been more than a site of entertainment. The Bollywood film industry is an essential component of South Asia's popular culture (Dudrah, 2006). It shapes values, identities, and everyday practices within India as well as across diasporic communities worldwide (Dudrah, 2012; Ganti, 2004; Takhar et al., 2012). Bollywood reflects, reproduces, and reconfigures social attitudes as a mass cultural form, often providing a lens through which audiences negotiate their understandings of gender, sexuality, and cinema (Kaur, 2002; Yadav and Jha, 2023). Existing scholarship has continually emphasized that cinema and its associated forms, such as songs, dance sequences, and visual spectacles, are not consumed passively but circulate as socio-cultural texts that audiences integrate into their lived realities (Clarke et al., 2024; Clarke and Hine, 2024; Fedorak, 2009; Louis and Chithra, 2024; Do Nascimento, 2019; Storey, 2014). Thus, Bollywood's representations of women have become one of the most engaging and contested issues (Kavya et al., 2025). Within this landscape, two key theoretical perspectives: the Reinforcement Theory of Motivation and the Madonna-Whore Dichotomy are integral in understanding how Bollywood's portrayals shape audience perceptions. The Reinforcement Theory of Motivation elucidates how repeated reward-punishment cues in media condition behavioral attitudes, while the Madonna-Whore Dichotomy reflects Bollywood's long—standing binary construction of “virtuous heroine” vs. “sexualized item girl.”

The portrayal of women in Bollywood films has historically been shaped by binaries that confined them to narrow roles (Gehlawat, 2015). From the 1950s onwards, Indian cinema distinguished between the heroine, seen as the virtuous, self-sacrificing woman, who embodies ideals of purity and respectability, and the vamp or “item girl” who is hypersexualized, transgressive, and ultimately excluded from narrative legitimacy (Datta, 2000; Narayanan, 2022). The item girl in particular, occupies a distinctive place in Bollywood's cultural imagination (Brara, 2010). She appears in musical sequences designed to attract attention and provide spectacle, but is seldom integral to the narrative. Moreover, the item girl's role contrasts sharply with that of the heroine, whose desirability is constructed around chastity, loyalty, and eventual validation by the male protagonist (Akhtar and Azmat, 2025; Pandey and Pandey, 2022). This binary, sometimes described in feminist literature as part of the Madonna-Whore Dichotomy (Hertler et al., 2023; Klein et al., 2024), continues to structure contemporary Bollywood films, including recent blockbusters such as Animal (2023) (Chakravorty and Bera, 2024; Dildar et al., 2024).

The present study investigates this heroine-item girl binary by combining quantitative analyse with qualitative theoretical interpretation to examine how audiences perceive and internalize the heroine-item girl divide present in Bollywood cinema. Its primary objectives are to examine whether (a) audiences perceive a difference in portrayal between the female lead and the item girl, (b) whether women identify more with the heroine than the item girl, (c) whether item songs are associated with changes in women's perceptions of reality, and (Sd) whether male actors' interactions differ toward these two types of female characters. However, the socio-cultural consequences of this bifurcation extend beyond the screen. By consistently framing women within the dual categories of the “good” heroine and the “bad” item girl, Bollywood cinema contributes to the moral policing of women's identities in Indian society and reinforces patriarchal gender codes (Habib, 2017; Mistri and Dasgupta, 2024). Such portrayals, thus, encourage audiences, particularly women, to internalize restrictive expectations about their identities, appearances, and sexual agency (Das and Raman, 2019). The heroine becomes the ideal that women are expected to emulate, while the item girl becomes a cautionary figure. She is perceived as a representation of transgression that invites public condemnation. These cinematic constructions resonate strongly with the everyday perceptions that women encounter; where notions of virtue, modesty, and purity remain deeply ingrained in social discourse (Kapoor, 2018).

Feminist film theorists have also provided important insights into these dynamics. The concept of the male gaze by (Mulvey, 2013) elucidates how cinematic representations position women as spectacles for male pleasure, often reducing them to objects of visual consumption rather than agents of narrative action. Several studies (Mittal and Gurpur, 2021; Purohit, 2019; Saeed et al., 2019; Shaikh and Azam, 2016) have extended these arguments, examining how the repetition of stereotypes in films limits the representation of women and reinforces broader patriarchal ideologies. At the same time, the study draws on the Reinforcement Theory of Motivation to theorize how repeated onscreen reward-punishment cues associated with heroines and item girls may condition audience attitudes. This theoretical link is used to support measurable survey items aligned with the study objectives. Its reward/punishment mechanism conditions the audience to internalize prevalent gender norms as natural and desirable. The theoretical grounding links behavioral psychology with feminist film theory, thereby strengthening the study's analytical framework.

Methodologically, the study employs a quantitative survey (*n* = 300) administered across multiple Indian states and NRI audiences, and analyzed using descriptive statistics, reliability testing, ANOVA, chi-square tests, Pearson correlations, and multiple regression analyses to test associations between women's onscreen portrayals and audience perceptions. To ensure methodological transparency, the study employed a structured 20-item questionnaire developed from prior literature on gender representation, objectification, and the Madonna-Whore Dichotomy. Items were grouped into four constructs: portrayal difference, relatability, influence on reality, and the male actor's behavior. These were measured using Yes/No/Maybe anchors, along with two closed-ended items and one open-ended item. The instrument underwent a pilot test with 30 participants to refine clarity and reliability before full-scale administration. The study, therefore, situates the dichotomy of the heroine and the item girl in Bollywood cinema within the larger sociological framework of South Asian popular culture.

Key empirical findings from the present dataset indicate that audiences distinguish between the heroine and the item girl (ANOVA and chi-square results were statistically significant), and the portrayal of the item girl is positively associated with perceived real-world impacts (Pearson *r* = 0.431, *p* = 0.004). The portrayal of the item girl emerged as the strongest predictor of behavioral perceptions in the regression model (*p* < 0.001). These results are interpreted as associations rather than causal effects due to the cross-sectional design. The findings elucidate that Bollywood's moral and visual binaries (such as the Madonna-Whore dichotomy) are internalize d by audiences. It reinforces patriarchal expectations of women's roles both onscreen and in everyday life.

Feminist sociology of media has long interrogated how gendered representations structure audience meaning-making, moral evaluation, and social categorization. Moreover, empirical research has increasingly shifted from asking whether sexualized media influence audiences to examining how audiences interpret, negotiate, and morally classify gendered representation. However, these empirically grounded debates remain disproportionately centered on Western media contexts, with limited quantitative audience research addressing how such classificatory processes operate within popular cinemas such as Bollywood. The present study addresses this gap by empirically examining audience differentiation between the heroine and the item girl as a sociological process of moral categorization rather than direct behavioral influence.

## 2 Theoretical framework

The study is grounded in the Reinforcement Theory of Motivation, which aids in examining how cinematic representations of women's characters in Bollywood influence audience perception. Rooted in B.F. Skinner's concept of operant conditioning, Reinforcement Theory of Motivation, states that individuals tend to repeat behaviors, and internalize attitudes that are socially rewarded, while avoiding those that are met with disapproval or sanctions (Gordan and Krishanan, 2014; Skinner, 1958). When this is applied to mass media consumption, it suggests that audiences are not merely passive recipients of onscreen portrayals, but they can adjust their perceptions based on the perceived rewards or punishments associated with certain representations (Klapper, 1963).

Applied to Bollywood cinema, the framework elucidates how audience perceptions of women are influenced by the repeated representation of two contrasting archetypes. The binary between the “heroine” and the “item girl” exemplifies such a reward-punishment mechanism in film narratives. Moreover, the heroine is portrayed as virtuous, modest, and worthy of respect, which aligns with the socially sanctioned ideals of femininity. However, the item girl is depicted as hyper sexualized and, transgressive, and serves as an objectified figure. Thus, through repetition, these portrayals operate as reinforcement cues. They condition audiences, particularly women, to internalize patriarchal expectations of behavior, and morality as natural and socially acceptable. These binaries implicitly indicate which forms of behavior are rewarded and which are disregarded within patriarchal social structures (Klapper, 1957).

The framework is conceptually developed from the Madonna-Whore Dichotomy, which is later expanded in feminist psychology (Bareket et al., 2018; Hertler et al., 2023; Klein et al., 2024). This dichotomy categorizes women into two polarized identities: the “Madonna”, who is the virtuous, pure, nurturing, and the “Whore,” who is sexualized, independent, and morally condemned (Young, 1993). In Bollywood cinema, this dichotomy manifests as the “heroine-item girl” binary. Here, the heroine embodies cultural virtue and emotional depth, while the item girl symbolizes temptation and moral transgression. The coexistence of these archetypes reproduces the psychological split between idealized and stigmatized femininity. Integrating the Reinforcement Theory of Motivation with the Madonna-Whore Dichotomy offers a dual explanatory model. The former explains how these portrayals shape perception (through behavioral conditioning) while the latter explains why women are divided into moral categories in the first place. Subsequently, they elucidate the process in which Bollywood's recurring depictions reward conformity to traditional gender roles and punish deviation. This theoretical synthesis further bridges feminist film theory and behavioral psychology. While feminist approaches such as Mulvey's male gaze critique how women are visually objectified in cinema, the Reinforcement Theory of Motivation provides a behavioral mechanism that explains how audiences internalize such visual codes over time. Thus, the study extends this discussion from representation to reception by associating cinematic imagery with audience cognition and belief formation.

The integration of the Reinforcement Theory of Motivation with the Madonna-Whore Dichotomy offers an explanatory framework that uncovers a mechanism-oriented understanding of media influence. While feminist film theory has strongly depicted how women are symbolically divided into morally celebrated, and marginalized categories, the questions surrounding how such divisions are cognitively sustained within audiences over time remain underexplored. Moreover, Reinforcement Theory complements this gap by examining how repeated exposure to “reward” representations (such as respect, narrative centrality, and moral legitimacy afforded to heroines), and “punishment” representations (such as objectification, narrative disposability, and vilification) can condition audience perceptions. Therefore, the framework extends existing feminist, and media-effects perspectives by offering a sociologically grounded explanation of how gendered binaries persist not only at the level of representation but through patterned audience evaluation and perception.

For female viewers, such dichotomous representations function as a conditioning medium, reinforcing the desirability of compliance with traditional gender norms while cautioning against transgression. For male viewers, reinforcement takes the form of associating masculine dominance and sexist behavior with positive outcomes. The theoretical framework provides the foundation for the study's research design. It explains the selection of variables (perception of the heroine and item girl, moral evaluation, audience identification, and influence of item songs). Therefore, examining audience perceptions through the Reinforcement Theory enables a deeper understanding of how media perpetuates or promotes gendered norms, and how these dynamics contribute to the broader socio-cultural impact of Bollywood cinema in contemporary India.

## 3 Literature review

Bollywood cinema occupies a crucial position in South Asian culture. It functions as a powerful socio-cultural text that shapes and reflects social values. The representation of women onscreen has been one of the most engaging and contested aspects of this cinema (Kaplan, 2012; Orwin, 2002). On the one hand, women have been revered as goddesses or inspirational figures, presented as embodiments of virtue, sacrifice, and respectability (Carroll, 1990; Doughman and Wael, 2025; Moodley, 2003). On the other hand, Bollywood films have simultaneously constructed women as objects of spectacle and desire, particularly through the trope of the “item girl” in songs (Sen, 2019). This duality exemplifies the Madonna-Whore Dichotomy elucidated in the theoretical framework, where women are divided into virtuous and transgressive categories. The duality reveals how Bollywood cinema facilitates both reverence and objectification, thus producing narratives that the audience internalize s as part of their cultural imagination (Tariq et al., 2021). Recent studies (Fatima and Islam, 2021; Habib, 2017) confirm that this division continues in contemporary cinema. It indicates the persistence of patriarchal reinforcement through popular media narratives.

### 3.1 The evolution of the heroine-item girl binary

From the 1950s onwards, Indian cinema established a clear binary between the virtuous heroine and her counterpart, identified as the vamp and later reimagined as the item girl (Kishore, 2014; Nijhawan, 2016). The heroine was written as pure, selfless, and chaste, and positioned as the moral center of the film. Meanwhile, the vamp or item girl was marked by sexual autonomy, moral ambiguity, and transgression. This division extended beyond narrative roles into the moral values attached to women themselves (Fox and Bailenson, 2009; Santoniccolo and Tommaso, 2023). The heroine represented the ideal Indian woman in films, whereas the item girl became a symbolic warning of what women should not be. Scholars have noted that this binary continues to structure mainstream films well into the present, with the heroine and item girl embodying contrasting identities of virtue and sexuality (Szurlej, 2017).

The heroine-item girl opposition has a longer genealogy within Indian cinema (Mukhopadhyay and Banerjee, 2021). It can be traced back to the classic “vamp—virgin” division in the 1950's and 1960's. The vamp's modern clothing, sexual openness, and association with nightclubs contrasted sharply with the heroine's chastity and sacrificial virtue (Dwivedi, 2017). With the decline of the vamp figure in the late 1980's, Bollywood gradually reintroduced her traits in the form of the “item girl” from the 1990s. Prevalent studies (Kaur, 2011; Narayanan, 2022; Shewade, 2023; Siddiqi, 2020) indicate three recurring archetypes across this lineage. The first is the vamp who embodies overt sexuality, the second is the dancer-by-profession whose livelihood justifies her visibility, and the third is the noble heroine whose sacrifices consolidate her virtue. These archetypes suggest how female characters have historically been compartmentalized into opposing moral categories in Bollywood cinema. This dynamic is visible in contemporary item songs in Bollywood cinema as well. The archetypes reflect the reward-punishment mechanism described by Reinforcement Theory of Motivation, where conformity to social virtue is rewarded through narrative approval and deviation is punished through exclusion or ridicule.

### 3.2 The Madonna-Whore dichotomy

The “heroine/item girl” binary can also be theorized through the Madonna Whore complex (Freud, 1910), which elucidates the portrayal of the “good/pious” woman vs. the “sexual/deviant” woman (Hertler et al., 2023). The former is typically coded as the moral ideal with domestic attributes, selfless, and worthy of male devotion. Meanwhile, the latter is framed as excessive, disruptive, and unsuitable for lasting relationships (Owen et al., 2007). Bollywood cinema reproduces this dichotomy through its parallel depictions of heroines and item girls. This translates psychological archetypes into cinematic conventions. Such repetition creates a conditioned response in audiences, which is consistent with the Reinforcement Theory of Motivation. Under this, moral approval of the heroine and disapproval of the item girl become habitual reactions. Such representations not only structure cinematic narratives, but also permeate into everyday perceptions about “good” and “bad” representations of female characters in films as well as in society.

Studies have elucidated that endorsement of this dichotomy is linked to patriarchal attitudes, victim-blaming, and greater tolerance of gender inequality, demonstrating its damaging social influence. The division also negatively affects men's mental health, as studies indicate that men who view nurturance, and sexuality as mutually exclusive report lower relationship satisfaction, and greater difficulty forming healthy emotional, and sexual bonds, (Hertler et al., 2023; Kahalon et al., 2019; Klein et al., 2024). When Bollywood reproduces this dichotomy, it intensifies these psychological divisions by normalizing them on a mass cultural scale. The heroine becomes the only legitimate object of respect and long—term affection, while the item girl becomes the object of momentary desire and social rejection. This cinematic reinforcement disseminates real-world constraints on women's autonomy, self-representation, and sexual agency, while also sustaining relational dynamics that are rooted in inequality rather than mutual respect. Hence, the dichotomy acts as both a narrative and behavioral framework that regulates female identity within Bollywood cinema.

### 3.3 Item songs as sites of objectification and spectacle

Item songs have currently emerged as a major cinematic device. They are often disconnected from the film's main storyline but included for commercial gain and mass appeal. These sequences feature women as “item girls”, and their performances are crafted for the visual pleasure of spectators. Studies have shown how item numbers are designed to maximize publicity, reinforce branding, and guarantee box-office success by foregrounding hyper sexualized femininity (Brara, 2010; Kapoor, 2018). Such portrayals rely on cinematic techniques through revealing costumes, choreographed bodily movements, objectifying camera angles, and voyeuristic lyrics. These align women's bodies with the concept of the male gaze. The woman is placed at the center of male gaze within these performances, thus reinforcing her construction as an object of desire (Mishra, 2021). From the perspective of the Reinforcement Theory of Motivation, these recurring portrayals reward the audience's visual pleasure while normalizing the moral distinction between the respectable heroine and the sexualized performer.

### 3.4 Audience perception and societal impacts

The integration of these cinematic portrayals into social life has significant consequences. Studies link the hyper sexualized representation of women in item songs to broader issues of gender stereotyping, moral policing, and even sexual violence in Indian society (Jain et al., 2019; Kulshreshtha and Jose, 2025; Patel, 2024). The absence of comprehensive sex education, along with the dominance of such binary portrayals, reinforces regressive attitudes toward women and perpetuates their objectification. Moreover, through the Reinforcement Theory of Motivation, audience perception can be understood in terms of the rewards or punishments associated with the on-screen portrayal of women in Bollywood cinema (Malone, 1975). Due to the reward or punishment mechanism, female viewers internalize cinematic binaries by embodying the socially sanctioned image of the virtuous heroine while distancing themselves from the stigmatized figure of the item girl. Therefore, it is integral to understand how women themselves perceive these dichotomous portrayals to assess the socio-cultural impact of Bollywood cinema.

The existing literature has empirically established that Bollywood cinema mostly relies on gendered stereotypes and moral binaries in its portrayal of women, and that audiences are not passive recipients of such binaries. Studies have documented normalization and symbolic consumption of female representation in Bollywood cinema. However, what remains underexplored is how these binaries operate as perceptual and moral categories through which audiences perceive femininity. Current audience research does not adequately explain the mechanisms through which repeated exposure may sustain such binary portrayals of female characters in Bollywood cinema. The present study, therefore, contributes beyond existing audience research by empirically examining the patterned differentiation and moral judgment that structure audience responses to female cinematic archetypes.

### 3.5 Research gap

Most existing scholarship has examined item songs and the heroine-item girl binary through textual or critical discourse analyses of films, focusing on cinematic techniques, lyrics, and historical tropes (Al-Maliki, 2025; Karsay and Johannes, 2018). The studies have been instrumental in exposing how gendered meanings are constructed and normalization at the levels of representation. However, the existing scholarship assumes the ideological effects of representation rather than empirically examining how Indian audiences actively perceive, interpret, and morally evaluate these female archetypes.

Existing audience studies within media sociology focus on broader questions regarding Bollywood cinema's influence, character identification, or general attitudes toward women. However, the factors behind the bifurcation of female characters, much observed in Bollywood cinema, remain underexplored. The empirical studies regarding the socio-psychological factors responsible for this bifurcation of the heroine-item girl in Bollywood cinema, thus, have not been explored in detail. Furthermore, the study takes into account underlying questions regarding this dichotomy, as merely a cinematic trope or whether it operates as a cognitive framework that dictates how audiences categorize notions of femininity, respectability, and desirability portrayed in films. Moreover, fewer studies have systematically examined how repeated exposure to such binaries may reinforce evaluative judgments through the process of reward/punishment and normalization.

The study, therefore, addresses this gap by shifting analytical attention from representation critique to audience reception and perception internalization. Furthermore, the study investigates how gendered cinematic binaries are recognized, differentiated, and normalization by audiences, by empirically examining audience responses to the heroine/item girl binary present in Bollywood cinema. Therefore, it maps patterns of audience perception by extending existing feminist critiques into an audience-centered sociological analyse of how gendered meanings circulate beyond the screen.

### 3.6 Research objectives

- To investigate whether there is a difference in the portrayal of the item girl as compared to the female lead in films.
- To examine whether women in real life relate more to the female lead as compared to the item girl.
- To determine whether item songs reflect women's perceptions of reality.
- To assess if there is a difference in the male actor's interaction with the item girl as compared to the female lead.

## 4 Method

### 4.1 Research design

The study adopts a descriptive and explanatory quantitative research design, integrated by qualitative interpretive analyse grounded in Reinforcement Theory and the Madonna-Whore Dichotomy, to investigate how audiences perceive the contrasting portrayals of the heroine and the item girl in Bollywood films, and how these perceptions may influence broader gender attitudes. This approach enables both the description of audience perceptions and the examination of statistical relationships among variables such as gender, relatability between the two female characters, and overall depiction of female characters. While most existing research on South Asian cinema relies on textual or discourse analysis, few studies have employed an empirical audience-centered approach. Therefore, by adopting a survey-based method, the study aims to provide empirical evidence of how young Indian audiences, who are the largest consumers of Bollywood, interpret and relate to these representations. Along with this, the methodological design of the study is theoretically informed by feminist media sociology and behavioral conditioning frameworks. The study conceptualizes it as a socially conditioned process shaped by repeated exposure to symbolic rewards and moral cues embedded in cinematic narratives. This theoretical orientation informs the choice of variables, response categories, and analytical focus on differentiation, evaluation, and moral judgment rather than attitudinal intensity.

Statistical analyses were conducted using IBM SPSS (Version 27), which included reliability tests, ANOVA, Chi-square, correlation, and regression analysis. The design ensures analytical rigor and alignment between theoretical constructs and empirical findings. The Reinforcement Theory and the Madonna-Whore Dichotomy support the construction of survey items, linking moral approval, relatability, and social attitudes to reward-punishment mechanisms in perception. Specifically, the dichotomy's core binary: virtue associated with the “Madonna” (heroine) and sexual transgression associated with the “Whore” (item girl) informed how the variables were conceptualized and interpreted within the survey. The study focused on representation-oriented variables rather than deeper cognitive, cultural, or media—environment factors, which could not be incorporated due to scope and feasibility constraints. These limitations are also acknowledged in Sec 7.

### 4.2 Sample and data collection

A non-probability convenience sampling method was used to recruit respondents based on their willingness to participate in the study. The survey targeted both college-going students and members of the working population across different Indian states, including Tamil Nadu, Andhra Pradesh, Karnataka, Kerala, Maharashtra, New Delhi, Jharkhand, West Bengal, Assam, and Nagaland and a small group of NRI respondents. This distribution was designed to ensure cultural and linguistic diversity among the respondents. Although the sample leans toward younger respondents due to a higher participation rate among students, this reflects Bollywood's primary viewing demographic. This makes the data contextually appropriate for the study's objectives.

Data were collected through a structured questionnaire administered in both online, and offline modes over a period of 3 months (November 2024 to January 2025). The instrument consisted of demographic questions and a section covering four main dimensions, including (a) portrayal difference, (b) relatability, (c) influence on reality, and (d) male actor's behavior. The questionnaire was developed using a theory-driven, literature-derived process in which survey items were constructed from recurring themes in prior research on Bollywood's gendered representation rather than adapted from existing validated scales.

The items were grouped into four conceptual dimensions, aligned with the research objectives. Items measuring audience perceptions of the heroine and the item girl were not taken from pre-existing scales; instead, they were designed based on conceptual patterns reported in prior studies on: (a) female representation in cinema (Chakraborty et al., 2017; Gupta-Cassale, 2000; Peter and Valkenburg, 2007); (b) objectification and item songs (Flynn et al., 2016; Kumar, 2017; Rodgers and Hust, 2018); (c) the Madonna-Whore dichotomy (Schulze et al., 2019; Schwichtenberg et al., 2019; Sinha and Ali, 2024); and (d) media-driven gender attitudes (Fischer and Greitemeyer, 2006; Khadilkar et al., 2022) to ensure transparency in measurement.

A drop-and-collect method was employed for the offline component to maximize participation and ensure more complete responses. Out of 350 responses collected, 300 valid responses were retained for analyse after screening for incomplete or inconsistent entries. The small group of NRI respondents (4%) was retained in the final dataset, as their inclusion offered additional insight into diasporic audience perspectives on Bollywood's gender portrayals. Due to the exploratory nature and descriptive statistical design of the study, these respondents were included in aggregate analyses but not treated as a subgroup. This yielded a strong response rate of 85%. Reliability testing of the instrument using Cronbach's Alpha (0.813) confirmed high internal consistency across items.

### 4.3 Instrumentation

The initial pool of items was generated from recurring constructs identified across these studies— such as virtue/respectability, sexualization, moral judgment, male validation, and real-life identification. These themes were used to construct original survey items, following a theory-driven scale development approach rather than modifying or reproducing existing measurement scales. The items were then aligned with the four research objectives of the present study to ensure conceptual relevance. Content validity was established by mapping each item to the Reinforcement Theory of Motivation and the Madonna-Whore Dichotomy, ensuring systematic measurement of reactions to “ideal woman” vs. “sexualized woman” portrayals. A pilot test was conducted with 30 participants to assess clarity, item reliability, and response consistency. Feedback from the pilot study led to minor revisions in wording and formatting to enhance item comprehension and reduce ambiguity before final administration. The final instrument consisted of 20 close-ended items, all measured using the anchors Yes/No/Maybe, except for items requiring a single close-ended response (Items 10, 14) or open-ended responses (Item 16). The items were grouped according to the study objectives as follows:

- Portrayal Difference (Items 4, 5, 7, 9): These items assessed audience perception of differences in appearance, behavior, and representation between the heroine and the item girl. Items were developed based on literature describing cinematic binaries and sexualization rather than adapted from pre-existing instruments.
- Relatability and Self-Identification (Items 10, 12, 13, 14): These items measured the extent to which audiences relate to either character in real life, reflecting the Madonna-Whore dichotomy and identity internalization frameworks.
- Influence on Reality and Social Perception (Items 11, 15, 18, 19, 20): These items captured perceptions of whether item songs influence women's identities, social judgment, and categorization in the real world, constructed from themes in media-effects research.
- Male Actor's Behavior and Gendered Interaction (Items 8, 17): These items evaluated perceived differences in how male actors treat the heroine versus the item girl, grounded in feminist film theory and reinforcement mechanisms.

Items 1-3 and 6 served as supporting contextual variables (frequency of item songs, attire-based differences, and behavioral commonalities) and contributed descriptive clarity. Items 1-3 and 6 served as supporting contextual variables (frequency of item songs, attire-based differences, and behavioral commonalities) and contributed descriptive clarity.

The decision to not employ Likert-scale measurements was a deliberate epistemological choice aligned with the study's theoretical focus on binary moral classification rather than attitudinal intensity. The heroine-item girl distinction in Bollywood cinema operates through categorical moral coding of virtue vs. transgression, rather than through gradated opinion. The Yes/No/Maybe response format was designed to capture categorical moral perceptions rather than nuanced attitudinal gradations. Given that the heroine-item girl dichotomy itself operates through binary moral classifications within popular culture, the response structure mirrors the sociological logic of the phenomenon under investigation. The aim was not to measure ideological complexity in the perception of female characters, but to identify patterned distinctions in audience evaluation.

### 4.4 Ethical considerations

The study adhered to the ethical guidelines for research involving human participants. Approval was obtained from the Institutional Ethical Committee for Studies on Human Subjects (IECH) at Vellore Institute of Technology, Chennai (Ref. No. VIT/IECH/CC/2024/34, dated March 12, 2025). All participants were informed about the purpose of the study, assured of confidentiality and anonymity, and provided their voluntary informed consent before participation. The study also complied with the participants' rights to withdraw at any stage without consequence.

### 4.5 Consent statement

The participants were informed about the purpose of the study, and assured of confidentiality, and anonymity. They provided their voluntary informed consent before completing the survey. Data were anonymized before analysis, and no personal identities were stored or shared.

## 5 Data analysis and results

For data analysis, the study employed statistical techniques in IBM SPSS (Version 27), including reliability test, ANOVA, Chi-square, correlation, and regression analysis. Descriptive statistical analyse was used to achieve the study's objective of comparing and analyzing audience perceptions of the female lead roles in Bollywood films and the women who perform as item dancers in Bollywood songs. A quantitative approach was selected to provide valid and accurate insights into how young audiences perceive these portrayals and the extent to which they internalize or reject the binaries presented onscreen. Along with the statistical analysis, the findings are interpreted through a theoretically informed analytical lens drawing on the Reinforcement Theory of Motivation and the Madonna-Whore Dichotomy. This interpretive approach is used to contextualize and explain observed quantitative patterns in audience responses, rather than constituting a separate qualitative dataset. More importantly, variations in audience evaluations are examined with regard to the symbolic reward and punishment structures embedded in Bollywood's cinematic representations, while differences between the heroine and the item girl are analytically understood through the moral polarity, which is integral to the Madonna-Whore Dichotomy. This theory-driven qualitative interpretation complements the quantitative findings by situating them within established sociological frameworks, without making claims to standalone qualitative analysis.

The survey instrument included relevant demographic information. This consisted of gender, age, employment status, marital status, and area of residence. Moreover, the perception items related to the portrayal of the heroine vs. the item girl, respondents' self-identification or relatability to each role, perceptions of how item songs influence views of women and gender relations, and interpretations of male actors' interactions with heroines vs. item girls. The data were cleaned, and coded before analysis. This ensured internal consistency and reliability of the variables used for interpretation. Subgroup analyses (e.g., gender, age group, or region) could not be conducted due to the limited sample sizes within each subgroup. All statistical tests were two-tailed, and significance was evaluated at the 0.05 level. The study, therefore, reports aggregate results only. Future research with larger stratified samples is recommended to test whether the observed patterns remain consistent across demographic groups.

### 5.1 Demographic information of the respondents

The demographic details of the participants are represented in the following Table 1.

**Table 1:** Demographic profile of respondents.

**Measures**	**Items**	**N (*n* = 300)**	**Percent**
Gender	Male	150	50.0
	Female	148	49.3
	Others	002	00.7
Age	17–25	242	80.7
	26–35	051	17.0
	36 and above	007	02.3
Employment status	Working	056	18.7
	Students	244	81.3
Marital status	Unmarried	275	91.7
	Married	025	08.3
Area of residence	South India	256	85.3
	North India	032	10.7
	NRI/outside India	012	04.0

Table 1 presents the demographic distribution of the sample. The gender distribution was nearly balanced, with 150 male respondents (50%) and 148 female respondents (49.3%), alongside 2 identifying as “other” (0.7%). The majority of participants (80.7%) were in the 17 to 25 years age group. This reflects the young profile of Bollywood's primary audience. Respondents aged 26 to 35 comprised 17%, while only 2.3% were aged 36 or above.

With regard to the employment status, 81.3% were students and 18.7% were working professionals. A large majority of respondents were unmarried (91.7%), and only 8.3% married. In terms of residence, most participants were from South India (85.3%), with smaller proportions from North India (10.7%) and the NRI/outside India (4%). These NRI responses were retained in the overall analyse to capture the diasporic audience perspective.

This demographic profile reflects a sample dominated by young South Indian audiences. This is a limitation that constrains the statistical generalizability of the findings across age groups and regional populations. However, this concentration is analytically significant as younger audiences occupy a structurally central position in Bollywood cinema's consumption, as they are the frequent viewers and digital circulators of Bollywood songs and visual culture (Statista Research Department, 2025). Their interpretations, therefore, provide insight into how cinematic binaries are received, normalized, and reproduced within a key demographic that actively forms the visibility and circulation of Bollywood cinema. Prior to participation, respondents were screened for regular exposure to Bollywood cinema and item songs, including self-reported familiarity with Hindi lyrics and representations of women, ensuring that the South Indian student sample understood and engaged meaningfully with the cultural texts under study. Although the findings cannot be extended to all age groups, they shed light on the perceptual frameworks operating within a generational cohort that plays an integral role in sustaining contemporary Bollywood cinema.

### 5.2 Reliability test

To assess the internal consistency and stability of the scale measurements, a reliability test was conducted. The Cronbach's Alpha value for this study is 0.813, which exceeds the threshold value of 0.7. This indicates good internal consistency (Table 2). Moreover, this indicates that the constructs used to capture audience perceptions of the heroine and item girl were stable and reliable, strengthening the validity of subsequent analysis. The relatively high alpha value confirms that the survey items effectively measure the underlying construct of audience perception toward gendered representations.

**Table 2:** Reliability analysis of constructs measuring audience perception.

**Scale/Construct**	**Cronbach's alpha**	**No. of items**
Audience Perception Scale	0.813	16

### 5.3 ANOVA (analysis of variance)

A series of one-way ANOVAs was conducted to examine whether audience perceptions differed significantly across variables related to the portrayal of the female lead and the item girl. One-way Analyse of variance (ANOVA) was employed to assess whether mean differences existed across audience evaluations on key perceptual dimensions. ANOVA is appropriate in this context because it allows comparison of group-level variation in averaged perceptual responses derived from categorical survey items, aligning with the study's objective of identifying patterned distinctions in audience evaluation rather than testing causal effects or predictive relationships. As shown in Table 3, significant group differences were found for all six variables: Commonality [*F*_(2, 297)_ = 3.33, *p* = 0.037, η^2^ = 0.347], Showing Women Differently [*F*_(2, 297)_ = 3.86, *p* = 0.022, η^2^ = 0.310), Compartmentalization of Women [*F*_(2, 297)_ = 3.10, *p* = 0.032, η^2^ = 0.324), Women in Real Life [*F*_(2, 297)_ = 5.11, *p* = 0.004, η^2^ = 0.349), Instances Faced [*F*_(2, 297)_ = 8.17, *p* < 0.001, η^2^ = 0.345), and Categorization by Attire [*F*_(2, 297)_ = 6.05, *p* = 0.002, η^2^ = 0.340). For the purpose of analysis, perceptual responses were grouped according to respondents' evaluations of the female lead and the item girl as distinct cinematic roles, with mean scores computed across relevant survey items. These groupings reflect differences in audience judgments of narrative legitimacy, moral positioning, and social validation rather than demographic segmentation or experimental conditions. The consistently large effect sizes indicate that audience responses differ markedly across the measured dimensions, reflecting stable patterns of perceptual differentiation between the heroine and the item girl. While these effect sizes fall within the moderate-to-large range, they should be interpreted as indicating the consistency of audience categorization rather than the intensity of belief or ideological internalization at the individual level.

**Table 3:** ANOVA Results showing differences in audience perception.

**Variable**	**SS Between**	**SS Within**	**df Between**	**df Within**	***F***	**Sig**.	**η^2^**
Commonality	222.670	419.050	2	297	3.330	0.037	0.347
Showing women differently	214.620	477.817	2	297	3.858	0.022	0.310
Compartmentalization of women	211.380	441.020	2	297	3.104	0.032	0.324
Women in real life	209.520	390.150	2	297	5.110	0.004	0.349
Instances faced	227.180	431.210	2	297	8.174	0.000	0.345
Categorization by attire	217.370	422.410	2	297	6.045	0.002	0.340

The findings address the first research objective by reflecting statistically significant differences in how audiences evaluate the portrayal of the heroine and the item girl. From an interpretive perspective, and without implying causality, these patterned differences are consistent with a theoretical interpretation that emphasizes moral differentiation within Bollywood cinema. The ANOVA results indicate an association between audience perceptions and the moral categorization of female characters, rather than evidence of socialization or behavioral conditioning.

Although the ANOVA results reflect moderate-to-large effect sizes, their sociological significance lies less in predictive strength and more in the consistency of audience categorization across representational dimensions. These effect sizes indicate that respondents effectively differentiate between the heroine and the item girl in patterned ways, suggesting the durability of binary perception rather than the intensity or depth of individual belief. From a sociological perspective, such consistency is meaningful because it reflects shared classificatory frameworks through which gendered characters are interpreted in popular Bollywood cinema. The magnitude of these effects reflects stable perceptual distinctions that align with entrenched representational reasons within Bollywood's narrative and visual conventions.

### 5.4 Chi-square analysis

A chi-square test is conducted to test homogeneity and goodness-of-fit among the variables of this study. Furthermore, the Chi-Square test analyses the statistical significance of two categorical variables used in this study, such as the portrayal of women in item songs and women as lead roles in films. From Table 4, it is observed that there is a significant association between the two categorical variables since the association value (*p*-value = 0.000) is found to be less than 0.05. This also confirms the first objective of the study, which states that audiences perceive, and Categorize the heroine and the item girl distinctly. In addition, the Pearson value (0.002) and value of the Likelihood ratio (0.008) are found to be significant, indicating that the study's variable has achieved homogeneity and goodness-of-fit. From an interpretive standpoint, without implying causality, this pattern is consistent with theoretical frame works that emphasizes binary moral classification in Bollywood cinema, such as the Madonna-Whore Dichotomy. The chi-square results, therefore, indicate an association between cinematic character type and audience categorization, rather than evidence of ideological conditioning or behavioral reinforcement. Therefore, the findings reflect patterned audience responses that align with prevalent representational binaries in Bollywood cinema.

**Table 4:** Chi-square test results on audience categorization of heroine and item girl.

**Test results**	**Value**	**df**	***p***	**Effect size**	**95% CI**
Pearson chi-square	16.723^a^	4	0.002	Cramer's V = 0.17	[0.09, 0.25]
Likelihood ratio	13.801	4	0.008		
Linear-by-linear association	12.486	1	0.000	ϕ = 0.204	[0.12, 0.29]
N of valid cases	300				

Variables: CAT, Categorical Judgement; PD, Perceived Difference.

^a^Effect sizes indicate small to moderate associations.

While the chi-square analysis reveals small-to-moderate associations between categorical variables, these relationships remain sociologically significant when interpreted at the level of shared cultural meaning rather than individual attitude formation. Modest associations can indicate the presence of widely circulating classificatory schemas that organize audience perception. In this case, the observed associations suggest that audience categorization of the heroine and the item girl follows recognizable moral and representational patterns, consistent with binary frameworks such as the Madonna-Whore Dichotomy. These findings should therefore be understood as reflecting patterned audience responses to cinematic cues, rather than as evidence of causal socialization or ideological conditioning.

### 5.5 Regression analysis

A multiple regression analyse was conducted to examine the relationship between behavioral difference and four predictors: portrayal of the item girl, perceived commonality between the female lead and the item girl, the perception that the two women are shown differently onscreen, and women's tendency to relate to these characters in real life, as shown in Table 5. The overall model was statistically significant and explained 61.2% of the variance in behavioral difference (*R*^2^ = 0.612, Adjusted *R*^2^ = 0.601, *F* = 54.82, *p* < 0.001). Among the predictors, portrayal of the item girl emerged as the strongest and most significant predictor (*B* = 0.423, β = 0.409, *t* = 7.779, *p* < 0.001), indicating that audience behavioral perceptions are shaped most powerfully by how the item girl is depicted. Relating to women in real life also had a significant effect (*B* = 0.232, β = 0.156, *t* = 3.039, *p* = 0.003). In contrast, commonality (*B* = 0.052, *t* = 1.106, *p* = 0.269) and the perception that the two women are shown differently onscreen (*B* = 0.086, *t* = 1.458, *p* = 0.146) were not statistically significant predictors in the model. The findings suggest that behavioral judgments are more strongly associated with the specific portrayal of the item girl than with general comparisons between female characters. From a theoretical perspective, without implying causation, this pattern is reflective of interpretations drawn from the Madonna-Whore Dichotomy, wherein moral evaluation is unevenly distributed across female character types. Similarly, when viewed through Reinforcement Theory as an interpretive lens, the findings indicate that Bollywood song portrayals are associated with evaluative distinctions rather than being received as neutral cinematic elements. Furthermore, these associations should be understood as perceptual alignments rather than evidence of behavioral conditioning.

**Table 5:** Regression analysis predicting behavioral perceptions.

**Model**	**R^2^**	**Adjusted R^2^**	**F-statistics**	***p*-value**
**Model Summary**				
1	0.612	0.601	54.82	0.000
**Model**		**Unstandardized coefficients**		**Standardized coefficients**	***t*** **-statistics**	***p*** **-value**
		**B**	**Std. Error**	**Beta**		
**Model Statistics**						
1	(Constant)	0.320	0.152		2.103	0.036
	Portrayal of item girl	0.423	0.054	0.409	7.779	0.000
	Commonality	0.052	0.047	0.257	1.106	0.269
	Showing women differently	0.086	0.059	0.178	1.458	0.146
	Relating to women in real life	0.232	0.076	0.156	3.039	0.003

Dependent Variable: Behavioral Difference.

The *R*^2^ value obtained in the regression Analyse is followed by cautious interpretation. Rather than indicating strong explanatory or causal power, this level of model fit is more appropriately understood as reflecting internal coherence among closely related perceptual variables used in the study. These predictors are conceptually overlapping, as they capture interrelated evaluative judgments about female character types rather than independent explanatory constructs. Because the predictors capture overlapping evaluative dimensions of cinematic representation, the model's strength lies in elucidating patterned alignment among audience perceptions rather than predicting behavioral outcomes. Sociologically, this coherence is significant as it reflects how different evaluative judgments form around particular character types, reinforcing the structured nature of audience interpretation without implying causal mechanisms or behavioral reinforcement.

### 5.6 Correlation analysis

Pearson correlation analysis examines the strength and direction of relationships among variables. Results indicate a strong positive correlation (*r* = 0.431) between the portrayal of the item girl and its impact on women in real life (Table 6), elucidating that such portrayals are significantly associated with audience perceptions of gender roles. Moderate correlations were also found between portrayals and the compartmentalization of women into binaries (*r* = 0.204) and the differing behaviors of the male actors toward the two women (*r* = 0.282). The results also confirm the second objective of the study, stating that women in real life relate more to the female lead, as compared to the item girl, with a positive correlation (*r* = 0.163, *p* = 0.005). These correlations reflect the audience's internalization of the Madonna—Whore dichotomy as the heroine is perceived as relatable and morally acceptable, while the item girl is linked to social judgment, behavioral caution, and moralized interpretations of sexuality.

**Table 6:** Correlation analysis of portrayals and audience perceptions.

**Variable**	**Correlation Statistics**	**Impact of the item girl's character on women in real life**	**Compartmentalization of women into two binaries in real life**	**Difference in the behavior of male actors toward the two women**	**Women relating more to the female lead**	**Division of women into these two categories in society**
Portrayal of female lead and item girl	Pearson correlation *p*-value N	0.431^*^ 0.004 300	0.204^**^ 0.009 300	0.282^**^ 0.000 300	0.163^**^ 0.005 300	0.117^*^ 0.042 300

^*^when *p* < 0.05, ^**^when *p* < 0.01, ^***^when *p* < 0.001.

These correlations indicate patterned relationships among audience perceptions rather than evidence of internalize d ideology or socialization. From an interpretive standpoint, the findings are consistent with theoretical interpretations that elucidate the moral differentiation in cinematic representations, such as the Madonna-Whore Dichotomy.

With respect to the fourth research objective, correlations involving the male actors' behavior suggest an association between narrative validation and audience perception. When interpreted through the Reinforcement Theory, this pattern can be understood as aligning with reward/punishment symbolism embedded within cinematic narratives. However, these findings do not indicate behavioral reinforcement. Rather, they indicate that audience perceptions co-vary with narrative cues related to validation and moral legitimacy.

Therefore, the statistical findings provide robust evidence that Bollywood cinema is consistent with gender binaries through a consistent reward-punishment mechanism. The Reliability analysis confirms stable measurement of audience perceptions, while the ANOVA and Chi-square establish significant categorical differences in how audiences perceive the heroine/female lead and the item girl. Moreover, Regression Analyse indicates a strong association between portrayals of the item girl, and behavioral judgments. This reflects how these portrayals are associated with real-life perceptions of women. Along with this, correlation analyse further indicates that the male actor's validation is associated with audience perceptions consistent with a reward-punishment mechanism. More importantly, these findings do not establish causal mechanisms of socialization or ideological conditioning. However, they suggest that audience perceptions are systematically aligned with existing gendered representations in Bollywood cinema. When interpreted through Reinforcement Theory and the Madonna-Whore Dichotomy as theoretical frameworks, the results indicate that Bollywood narratives are consistent with the moral bifurcation of female characters and symbolic reward-punishment structures. The use of multiple statistical techniques enhances internal coherence and analytical robustness, thus allowing the findings to be situated within sociological discussions of gender representation.

## 6 Discussion

The study employs a quantitative approach, supported by qualitative theoretical interpretation, to investigate the perception of Indian audiences toward the representation of women in Bollywood cinema. It particularly contrasts the portrayals of the female lead and the item girl in Bollywood. The demographic balance of male and female participants reinforces the reliability of the findings, showing how both genders perceive these cinematic representations. Through the Reinforcement Theory of Motivation, the results suggest that Bollywood cinema produces clear binaries in the representation of women. Most importantly, the representation is amplified through the dichotomy between the female lead and the item girl. The findings indicate how the Madonna-Whore Dichotomy framework operates as a psychological and cultural tool in understanding the bifurcation of female characters in Bollywood cinema. It is associated with rewarding purity and punishing sexual expression. These representations reinforce patriarchal gender norms by indicating which forms of femininity are socially validated/rewarded and which are punished. The findings are elucidated in detail through the following themes. The study contributes to feminist audience research by empirically examining how moral categorization and symbolic differentiation operate within audience responses toward gendered cinematic roles.

### 6.1 Reinforcement of gendered binaries

The first objective investigated whether there is a difference in the portrayal of the item girl as compared to the female lead/heroine. The ANOVA and Chi-square tests confirm that audiences perceive these roles as sharply distinct. This reflects established cinematic codes that place the heroine as virtuous, modest, and morally centered, while marginalizing the item girl to sexual deviance. When examined through the combined theoretical frameworks, the female lead is associated with behaviors that are socially rewarded, such as chastity, respectability, and moral virtue. Meanwhile, the item girl embodies traits that are socially punished, such as sexual deviance or flamboyance. At the same time, the statistical findings add to the supposition that such binaries are uniformly internalize d as explicit moral beliefs. The consistency of audience differentiation suggests recognition of representational codes rather than conscious ideological endorsement, indicating that the Madonna-Whore Dichotomy functions as a classificatory framework through which femininity is routinely evaluated. This form of recognition is itself a mechanism of reinforcement, as repeated categorization normalizes patriarchal distinctions even in the absence of deliberate acceptance. This argument is further validated by Connell (1990), elucidating how gender ideologies are maintained through oppositional categories that regulate femininity. This defines some forms as legitimate and others as deviant. Accordingly, Bollywood cinema exemplifies these oppositional categories by consistently dividing women into these two binaries of the female lead and the item girl (Khan and Taylor, 2018). This is associated with audience perceptions of femininity through a reward-punishment lens. However, the findings suggest that audiences engage with this dichotomy pragmatically, drawing on it as a familiar interpretive framework to make sense of cinematic narratives rather than as a rigid moral code that is applied uniformly to women in general. The consistent differentiation observed across statistical analyses indicates that audiences recognize and reproduce these representational distinctions when evaluating characters within films, particularly in relation to narrative legitimacy, respectability, and moral positioning. This nuance refines feminist critiques by showing that the persistence of the Madonna-Whore binary operates through cultural familiarity and narrative convention, whereby repeated exposure normalizes these distinctions even when their moral authority is not consciously articulated or explicitly endorsed. In this way, the dichotomy continues to structure audience perception by guiding interpretation and evaluation, rather than by demanding overt ideological commitment. Moreover, this binary representation not only reflects but also sustains patriarchal structures, reinforcing the perception of the female lead as an aspirational model and the item girl as a cautionary figure (Ahuja and Pundir, 2022). Thus, through the reproduction of these stereotypes, Bollywood cinema continues the normalization of restrictive gender roles.

### 6.2 Audience identification and relatability

The second objective examined whether women in real life relate more to the heroine as compared to the item girl. Correlation analyse reflects this distinction, showing that young audiences, particularly women, report strong identification with the female lead/heroine archetype. The pattern reinforces the Madonna-Whore archetype as a social script. The audiences associate the heroine's moral traits with ideal qualities and the item girl's sexualized identity with threat. The finding challenges deterministic interpretations of item songs as uniformly or automatically influential. Instead, the results suggest that audience responses depend largely on how these songs morally frame the female character, rather than on visual spectacle or sexualized imagery alone. In this sense, item songs influence perception not merely through visibility, but through the values and meanings attached to the portrayal. This inclination reflects the ways reinforcement mechanisms are associated with everyday perceptions. The female lead's traits are culturally coded as desirable, and thus, audiences are socially rewarded for aligning with the same traits. Likewise, the item girl's traits are considered socially undesirable; hence, audiences tend to avoid these traits, which are socially discouraged. From a sociological perspective, audience identification reflects what (Bourdieu, 2018) terms habitus. This is a system of dispositions shaped through repeated cultural exposure. However, the non-significance of “commonality” in earlier analyses indicates that audience identification is not primarily shaped by shared experience or perceived similarity with the female character. Instead, identification appears to be guided more strongly by symbolic moral positioning, where characters are evaluated based on cultural ideas of respectability and legitimacy. This finding refines the application of habitus in media contexts by showing that moral meaning, rather than experiential closeness, plays an integral role in shaping audience dispositions. Therefore, by repeatedly rewarding the heroine's conformity and punishing the item girl's transgression, Bollywood contributes to the internalization of gender norms within audience dispositions (Banaji, 2013). The Reinforcement Theory of Motivation elucidates why the audience is more willing to relate to the heroine, as her archetype secures legitimacy and validation. Meanwhile, the item girl's archetype invites social disregard. This selective identification indicates that patriarchal ideology is reflected not only through cinematic narrative but also through audience emotional alignment with virtue vs. transgression. It reinforces the idea that conformity to patriarchal expectations is both safer and more socially rewarding (Becker, 1999).

### 6.3 Item songs and the punishment mechanism

The third objective examined whether item songs reflect women's perceptions of reality. Regression analyse shows that two predictors: (1) the portrayal of the item girl and (2) women's tendency to relate to characters in real life, were significant predictors of behavioral perceptions, whereas commonality and the perception that the two women are shown differently onscreen were not statistically significant. This indicates that audience behavioral responses are shaped by specific moralized portrayals rather than general comparisons between the two female characters. Thus, it is the moral framing of the item girl and not merely visual differences that influence behavioral perceptions. This suggests that these representations are associated with more than just commercial entertainment. Instead, the songs operate within a reinforcement framework, celebrating the female lead and marginalizing the item girl's archetype. The finding challenges deterministic views that item songs exert a uniform influence on audiences. Instead, it suggests that their impact depends largely on how the female character is morally framed within the narrative, rather than on visual spectacle or sensual imagery alone. Audience responses, therefore, appear to be shaped by evaluative meanings attached to the portrayal, not merely by visibility or performance. Moreover, audiences tend to relate these cinematic representations to perceptions of women's identities. This dynamic reflects what Foucault, 2023 describes as disciplinary power. Here, systems of reward and punishment regulate behavior by defining what is socially acceptable and what is deviant. Item songs perform this disciplinary function through the Madonna-Whore dichotomy. They simultaneously eroticizes, and shame female sexuality, while celebrating desirability and denying agency. Through their exaggerated focus on bodily spectacle and male-gaze-driven choreography, item numbers reflect to the audiences the boundaries of acceptable femininity (Shah and Cory, 2019). It also reflects the grounds on which female sexuality is acceptable onscreen for the production of the masses. Moreover, this association does not suggest direct behavioral conditioning. Instead, it highlights how symbolic punishment works through narrative exclusion and moral ambiguity, rather than through clear or explicit sanctions. The item girl is thus visible within the narrative but denied moral legitimacy or lasting recognition. The archetype of the item girl, thus, contests these grounds and limits the representation of female sexuality and autonomy onscreen. The cinematic punishment mechanism ensures that the item girl's transgression is visually celebrated but socially condemned. This reaffirms the dichotomy between sacred and profane femininity.

### 6.4 Male validation as a reinforcement tool

The fourth objective examined whether there is a difference in the male actor's behavior toward the female lead and the item girl. Correlation findings confirm that audiences associate the female lead with romantic commitment, respect, and social approval from male leads, whereas the item girl is seen as lacking such legitimacy. Instead, the archetype is used as a temporary spectacle to advance the plotline or increase viewership, after which the character's utility is discarded, which is associated with a reductive or transgressive framing of female roles in cinema. Moreover, the argument on hegemonic masculinity by Connell and Messerschmidt (2005) strengthens this finding. Hegemonic masculinity operates as a cultural standard that not only privileges certain masculinities but also polices acceptable femininities (Schippers, 2007). Within this framework, male characters are associated with reinforcement of perceptions regarding legitimate forms of femininity. Their validation of the heroine is associated with the perception of socially sanctioned femininity. Meanwhile, their rejection of the item girl is associated with perceptions of deviance. Therefore, male validation is associated with the operation of both the Reinforcement Theory of Motivation and the Madonna-Whore dichotomy. This is because masculinity functions as the ultimate intermediary of feminine representation. It aligns with feminist media critique of the male gaze, wherein female visibility is conditioned/designed by male approval. However, the male validation does not operate as a single or absolute force shaping audience perception. Instead, it functions as a symbolic mediator that signals which female characters are granted narrative legitimacy and social approval. Audiences appear to use this validation as a cue to interpret respectability, commitment, and moral worth within the cinematic story.

The findings indicate that Bollywood cinema operates as a cultural apparatus where gender is reinforced through binary representations of virtue and transgression. Interpreted through the Reinforcement Theory of Motivation, the female lead's socially sanctioned traits are rewarded through narrative centrality and male validation, while the item girl's sexual presence is punished. The regression results further support this by showing that behavioral perceptions are shaped primarily by moralized portrayals of the item girl and by whether audiences relate to these portrayals in real life, rather than by general comparisons between the two characters. These findings suggest that reinforcement operates through perceptual alignment rather than direct behavioral conditioning, thereby refining the explanatory scope of reinforcement-based media theories. Moreover, these dynamics intersect with hegemonic masculinity, reflecting how male validation serves as a gatekeeper of women's identities and representation. From a feminist media perspective, these representations also commodify, and constrain female characters, thus reproducing the male gaze. It positions female characters as objects of spectacle rather than agents of action. Thus, item songs epitomizes this disciplinary cycle, sexualizing women for viewership while reaffirming the heroine as the moral ideal. This highly narrows the space for alternative representations of femininity. The implications also extend beyond cinema into everyday life, where these binaries are reproduced through the digitalization of item songs and films today. Therefore, the female representation through Bollywood cinema not only extends to cinematic stereotypes such as the Madonna-Whore Dichotomy but also perpetuates broader structures of gender inequality.

## 7 Limitations

The study includes several limitations that should be acknowledged. Firstly, the survey sample is dominated by young South Indian respondents, primarily students, which restricts the generalizability of the findings to older, rural, or more diverse populations. Secondly, the study employs a quantitative cross-sectional design, which identifies associations but does not establish causal relationships. Thirdly, self-reported responses may be influenced by social desirability bias, especially given the moral and gendered nature of the questions. Additionally, although the study draws on the Reinforcement Theory of Motivation and the Madonna-Whore Dichotomy to interpret audience perceptions, these frameworks do not fully capture the broader psychological, cultural, and contextual factors, such as personal beliefs, prior media exposure, and socio-cultural background, that also shape perception but were not included due to resource and scope constraints.

Furthermore, the use of convenience sampling may limit the generalizability of the results, as participants were selected based on ease of access rather than probability-based methods. With the sample concentrated within specific regions, and age groups, it may not fully represent the wider Indian population. Nevertheless, the demographic patterns observed, such as age, gender, and region, show internal consistency within the accessible sample, supporting the preliminary validity of the findings. Although convenience sampling cannot overcome representational limitations, the statistical analyses still provide foundational insights that can guide more comprehensive future research. While Cronbach's Alpha demonstrates internal consistency, the study does not claim full psychometric construct validation. The items were developed through theory-driven operationalization of concepts derived from feminist media theory and reinforcement-based behavioral frameworks.

Future studies could use mixed-methods designs, probability sampling, or qualitative interviews to explore how diverse populations interpret and negotiate gendered portrayals in Bollywood, and how contextual factors, including cultural background, media environment, and prior experiences that shape audience perceptions more holistically.

## 8 Conclusion

The study examines audience perceptions of the female lead and the item girl in Bollywood cinema, interpreting these portrayals through the combined lenses of Reinforcement Theory and the Madonna-Whore Dichotomy. Findings reveal that viewers clearly distinguish between the two archetypes: the heroine is associated with virtue, modesty, and relatability, while the item girl is positioned within the domain of sexual spectacle and moral transgression. This contrast reflects how Indian popular culture continues to reproduce the sacred-vs.-profane division of femininity, rewarding purity while punishing autonomy. Moreover, the results suggest that item songs significantly influence audience perceptions of women, shaping behavioral and moral expectations beyond the cinematic screen. Within the framework of Reinforcement Theory, the heroine is socially rewarded through validation, respectability, and narrative centrality, whereas the item girl is symbolically punished through marginalization and objectification. By extending this framework to the Madonna—Whore Dichotomy, the study shows how patriarchal ideology operates through both cinematic representation and audience reception, reinforcing gendered binaries as moral truths.

## Data Availability

The raw data supporting the conclusions of this article will be made available by the author, without undue reservation.
