# Supplementary material for: Representations of female characters in Bollywood cinema: stereotypes, audience perceptions, and societal impacts
Source: Front Sociol. 2026 Feb 12;10:1694300. doi: 10.3389/fsoc.2025.1694300 (PMC12935684; doi:10.3389/fsoc.2025.1694300)
Supplement: Supplementary file 2 [file Table_2.docx]

**Representations of Female Characters in Bollywood Cinema: Stereotypes, Audience Perceptions, and Societal Impacts**

DEMOGRAPHIC DETAILS

AGE:

GENDER: a) Male b) Female c) Others

WORKING/STUDENT: a) Working b) Student

EDUCATIONAL QUALIFICATIONS:

CURRENT CITY:

LANGUAGES SPOKEN:

ETHNICITY:

MARITAL STATUS: a) Married b) Unmarried

SECTION – I

Q1. Is there an increase in the number of item songs in Bollywood?

1. Yes b) No c) Maybe

Q2. Do you find any point of interest/uniqueness in the attire of the female lead?

1. Yes b) No c) Maybe

Q3. Is there any point of interest/uniqueness in the attire of the item girl?

1. Yes b) No c) Maybe

Q4.  Do you find any difference in the way the item girl is portrayed as compared to the female lead?

1. Yes b) No c) Maybe

Q5.  Do you find any difference between the behaviour of the female lead and the item girl?

1. Yes b) No c) Maybe

Q6. Is there any point of commonality between the behaviour of the female lead and the item girl?

1. Yes b) No c) Maybe

Q7. Are the two women shown differently in the films?

1. Yes b) No c) Maybe

Q8.  Does the male actor behave differently with the two women in the film?

1. Yes b) No c) Maybe

Q9.  Do you think there is a compartmentalization of these two women’s characteristics in the songs?

1. Yes b) No c) Maybe

Q10.  Which women do you relate more to, in real life?

1. Heroine/Female Lead b) Item Girl

Q11.  Do you think item songs impact women in real life?

1. Yes b) No

Q12.  Do you find characteristic traits/behaviours of the heroine in your real life?

1. Yes b) No

Q13.  Do you find any characteristic traits/behaviours of the item girl in your real life?

1. Yes b) No

Q14.  Which attire do you relate to in real life?

1. Heroine’s/Female Lead’s attire b) Item Girl’s attire

Q15. Do you think society divides women into two divisions in real life too?

1. Yes b) No

Q16. What are some of the terms/slangs associated with item girls in songs? (write as many as you are aware of) __________________________________________

Q17. Have you ever been addressed with these terms in real life by any individual?

1. Yes b) No

Q18. Are women also categorized as good or bad based on their behaviours/attires in real life?

1. Yes b) No

Q19. Have you faced any instances where you have been categorised in either of these categories based on your attire?

1. Yes b) No

Q20. Do you think you also categorize women based on their attire or mannerisms?

1. Yes b) No
